# Prediction of carbon emissions from public buildings in China’s Coastal Provinces under different scenarios ——A case study of Fujian Province

**DOI:** 10.1371/journal.pone.0307201

**Published:** 2024-07-23

**Authors:** Yanyan Ke, Rui Fan, Yan Yang, Pingying Wang, Jiarui Qi

**Affiliations:** 1 College of Harbour and Coastal Engineering, Jimei University, Xiamen, China; 2 Xiamen Key Laboratory of Green and Smart Coastal Engineering, Xiamen, China; University of Agriculture Faisalabad, PAKISTAN

## Abstract

With the rapid pace of industrialization and the increasing intensity of human activities, the global climate change and energy crisis have reached a heightened level of severity. Consequently, achieving an early peak in carbon emissions has become an imperative in addressing this pressing issue. Particularly, coastal provinces, known for their developed economies, high population density, and substantial building energy consumption, have emerged as significant contributors to carbon emissions. Notably, public buildings, serving as critical constituents of the construction industry, possess immense potential for both energy conservation and emissions reduction. In light of this, the present study focuses on Fujian Province, situated along the coast, and constructs a carbon emission estimation model for public buildings based on the Kaya identity. This model takes into account various factors specific to Fujian Province, including population characteristics, economic conditions, tertiary industry development, public building area, and energy consumption. Through scenario analysis, the study projects that the year of peak carbon emissions for public buildings in Fujian Province is estimated to be 2030, 2035, and 2040 under low-carbon, baseline, and high-carbon scenarios respectively. The corresponding peak carbon emission levels are anticipated to reach 23.62 million t, 24.18 million t, and 24.76 million t CO_2_. Lastly, based on local policies and actual conditions, the study proposes a set of policy measures and feasible approaches tailored to Fujian Province, aiming to achieve an early peak in carbon emissions.

## Introduction

In recent years, the global issue of climate change has gained increasing prominence due to its profound detrimental effects. In recognition of the imperative to curb greenhouse gas emissions and address global climate change, the United Nations General Assembly ratified the United Nations Framework Convention on Climate Change as early as May 1992. Subsequently, in December 2015, the Paris Agreement was adopted by 195 countries during the 21st Conference of the Parties to the UN Framework Convention on Climate Change, outlining measures for global climate change mitigation beyond 2020. During this pivotal conference, China made its own commitments to reducing emissions, including a target to reach the peak of carbon dioxide emissions by around 2030 and strive for an earlier attainment.

Numerous studies have demonstrated that energy consumption is particularly high within key sectors such as manufacturing, construction, and transportation, with the construction sector representing a significant proportion. According to the China Building Energy Consumption Research Report (2022) issued by the China Building Energy Efficiency Association, the carbon emissions proportions for building materials production, construction, and operation and maintenance after completion stand at 28%, 1.0%, and 21.6% respectively. Notably, the entire building process accounts for 45% of China’s total energy consumption and contributes to 50.6% of the nation’s overall carbon emissions. Undoubtedly, the construction industry in China substantially contributes to energy consumption. Over the past few decades, China’s industrial processes have accelerated, and with the sustained economic growth, the construction industry has developed significantly. As indicated in the Statistical Yearbook published by the National Bureau of Statistics, in 2021, the housing construction area in China reached 15.75 billion square meters, while the completed housing area amounted to 4.1 billion square meters, with the total output value of the construction industry reaching 29.3078 trillion CNY [1]. However, this rapid expansion of the construction industry has led to escalating carbon emissions during the production process, resulting in ecological degradation, particularly evident in global climate change and the energy crisis.

Coastal provinces have emerged as significant contributors to carbon emissions due to their developed economies, large population bases, and expansive public building areas. Between 2005 and 2019, these coastal areas accounted for 45%-50% of the national total, which poses a significant challenge to China’s dual-carbon goals. Fujian Province holds the distinction of being the first national ecological civilization pilot zone, having made notable strides in energy conservation and emission reduction during the 13th Five-Year Plan. Despite this progress, the area occupied by public buildings in Fujian Province has steadily increased, and their energy consumption per unit area remains notably high compared to residential buildings by 5–15 times [2]. This is 1.5–2 times higher than similar buildings in developed nations such as Europe [3]. To realize the goals outlined in the 14th Five-Year Plan and the dual-carbon objective, while also offering insights for other provinces, it is imperative to examine future trends in carbon emissions from public buildings in Fujian Province in conjunction with existing energy-saving policies. According to the Fujian Statistical Yearbook, water, electricity, gas, heating, and cooling represent the primary sources of building energy consumption. Given that public buildings account for one-third of China’s construction sector and continue to grow alongside urbanization and rising living standards, they present significant opportunities for energy conservation and emission reduction. Therefore, research on public buildings’ carbon emissions is crucial to achieving China’s carbon peak goal by 2030. To provide effective suggestions for energy saving, emission reduction, and sustainable development of public buildings in coastal provinces, this paper takes Fujian Province as a representative case study and raises the following three questions:

1. What factors influence carbon emissions from public buildings in Fujian Province?
2. Can the carbon peak goal by 2030 be achieved based on the current situation?
3. How do carbon emissions and the carbon peak situation of public buildings in Fujian Province vary under different scenarios?

To address these questions, the study aims to predict the carbon peak scenario of public buildings in Fujian Province by utilizing relevant statistical data, analyzing the key factors influencing carbon emissions, employing the Kaya Identity and STIRPAT model to forecast the time and peak value of future carbon peaks, and conducting scenario analysis to compare carbon peak values under different scenarios. Based on these findings, appropriate policy recommendations can be formulated to achieve the carbon peak goal as early as possible.

This study offers innovative contributions in multiple aspects. Firstly, it selects Fujian Province as a representative case study among coastal provinces, investigating the factors that impact carbon emissions from public buildings in the region. Consequently, the study can serve as a reference for research on similar topics in coastal provinces and bridge the research gap regarding carbon emissions from public buildings in such areas. Secondly, in terms of research methodology, the study combines the Kaya Identity and STIRPAT model to construct a comprehensive carbon emissions prediction model that considers multiple influencing factors. Additionally, scenario analysis is employed to forecast the carbon peak situation under diverse scenarios. Finally, from a research focus perspective, unlike existing studies that primarily concentrate on the national level, a single city, or specific buildings within a city, this study specifically focuses on public buildings in coastal provinces, providing theoretical support and offering policy references for achieving energy conservation, emission reduction, and green development in public buildings within these coastal provinces.

The remainder of the paper is organized as follows: Section 2 provides a literature review; Section 3 introduces the research methodology; Section 4 presents the analysis and results; Section 5 discusses the carbon peak under different scenario modes, and Section 6 concludes the research and offers policy recommendations.

## Literature

In light of the alarming environmental degradation and the substantial contribution of carbon emissions from public buildings to overall building-related emissions, scholars have dedicated considerable attention to researching this area. Presently, studies on carbon emissions from public buildings predominantly focus on influencing factors, carbon emission predictions, and lifecycle carbon emissions assessments. For instance, Jiang utilized data spanning from 1996 to 2008 as a sample to provide long-term predictions, analyze influencing factors, and evaluate the potential for carbon emission reduction and associated costs in China’s large-scale public buildings. Correspondingly, emission reduction pathways were proposed [4]. In a similar vein, Hua et al. analyzed the proportion of carbon emissions contributed throughout the lifecycle of public buildings. Their findings emphasized the significance of energy consumption reduction during the operational stage as the primary measure for controlling and mitigating carbon emissions from public buildings [5]. Jia et al. used Henan Province as a case study to calculate and analyze the ecological footprint from 1983 to 2006, and summarized the relevant influencing factors [6]. Furthermore, Du et al. investigated the spatiotemporal characteristics of carbon emissions from public buildings across 30 provinces in China during the period of 2008 to 2019. Their analysis revealed that population, urbanization rate, per capita GDP, green building index, and industrial structure exhibited spatial correlation and dissimilar impacts on carbon emissions from public buildings [7]. Similarly, Wang et al. employed the LMDI method to construct a macro carbon factor decomposition model for public buildings in China. The analysis demonstrated that reducing carbon emission coefficients and energy intensity served as the main driving factors in inhibiting the growth of carbon emissions from public buildings [8].

The advancements in research pertaining to building carbon emissions have greatly benefited from the utilization of various prediction models. Prominent among these models are the Kaya identity equation, the IAMC model, the IPAT model, the LEAP model, the STIRPAT model, and the LMDI model. Among them, the STIRPAT model stands out as the most extensively employed. In an evaluation conducted by York et al., the IPAT identity equation, IMPACT identity equation, and STIRPAT model were scrutinized, leading to the conclusion that the STIRPAT model provides a more accurate explanation regarding the driving sensitivity of factors influencing carbon emissions [9]. Johannes Lohwasser et al. asserted that the STIRPAT framework is commonly employed for assessing the impact of human activities on environmental outcomes, with its non-standardized form reflecting the slope of the relationship between the dependent variable and the considered predictive variables, thus effectively capturing the relationship between variables [10]. Lin et al., using Monte Carlo simulation, identified the factors influencing China’s carbon emissions and deduced that our country’s low-carbon transformation efforts should primarily focus on energy conservation while also incorporating the development of clean energy [11]. Zhang et al. based on the Kaya identity equation, reached the conclusion that the economic output effect and population size effect constitute the primary contributing factors to carbon emissions growth in Xinjiang [12]. Cai analyzed the driving forces behind the expansion of building energy consumption through the employment of the STIRPAT model, revealing that population, urbanization, building area, living standards, and building energy efficiency serve as key influencing factors in building energy consumption [13]. Ma argued that a well-established theoretical foundation has been formed for predicting future carbon emissions peak based on the IPAT model series (Kaya identity equation). Accordingly, the Kaya identity equation can be utilized to forecast carbon emissions peak and subsequently propose targeted policies for energy conservation and emission reduction [14]. AL-Mulali et al. employed the STIRPAT model to analyze and investigate the relationship between carbon dioxide emissions, urbanization, and energy consumption in various countries [15].

With the deepening research on building carbon emissions, many scholars have proposed the view that different climates and regions have a significant impact on carbon emissions, thus initiating research on building carbon emissions in different regions. She conducted a case study on a public building in Xiamen based on the theory of life cycle assessment, identifying key emission reduction periods throughout the building’s life cycle in regions with hot summers and cold winters [16]. Ke et al. conducted carbon peak prediction research on residential buildings in coastal provinces, using Fujian Province as an example. They utilized scenario settings to determine the carbon peak situation under different scenarios and proposed feasible suggestions [17]. Geng et al. studied the carbon emissions of urban residential buildings in the Guangdong-Hong Kong-Macao Greater Bay Area from a life cycle perspective, providing data references and recommendations for the low-carbon development path of urban residential buildings [18]. Wu et al. proposed a standardized framework for estimating indirect building carbon emissions within the boundaries of various local climate zones (LCZ). By linking building carbon emissions to LCZ, they determined the emission factors for different LCZ categories in Shanghai [19].

In summary, recent literature studies suggest that the utilization of the STIRPAT model and scenario analysis method is highly accurate in predicting the peaking of carbon emissions. However, limited research has been conducted on carbon emissions specifically from public buildings in Fujian Province. Hence, this study aims to address this gap by constructing the STIRPAT model based on the Kaya identity equation and employing scenario analysis to compare carbon peaks under different conditions. The objective is to explore the future prospects and peak research of carbon emissions from public buildings in Fujian Province, offering relevant recommendations to facilitate early carbon peaking in the province and establish a solid foundation for achieving carbon neutrality in a timelier manner.

## Research method

### Determination of influencing factors

This study characterizes and constructs the Kaya identity equation for the carbon emission intensity of public buildings in Fujian Province, drawing on an analysis of literature [10–34] and the current situation of carbon emissions. Six categories of influencing factors are selected, namely population, economy, development status of the tertiary industry, area of public buildings, unit energy consumption, and unit carbon emissions. To measure population, the total population is considered as a crucial indicator since carbon emissions closely relate to human activity. The study identifies three economic indicators—per capita GDP, the proportion of the tertiary industry, and the economic activity intensity of public buildings (i.e., the ratio of the total area of public buildings to the total GDP of the tertiary industry)—as crucial measures of economic prosperity. Energy efficiency is evaluated by including both per unit area energy consumption of public buildings and the total carbon dioxide emissions from unit energy consumption. Building area expansion has been identified as a direct cause of the increase in carbon emissions from public buildings in China. Specifically, Fujian Province has witnessed an average annual growth rate of approximately 15% in completed construction area, with a complete public building area of 169.5033 million square meters in 2010 and 415.6276 million square meters in 2020. Similarly, the total carbon emissions from public buildings increased from approximately 15.33272 million tonnes of standard coal in 2010 to 22.81207 million tonnes of standard coal in 2020, with the expansion of public building area being attributed as the primary driving factor. Therefore, this study considers the public building area in Fujian Province as one of the key influencing factors affecting variations in carbon emissions from public buildings.

### Predictive model

#### Kaya identity equation

Japanese scientist Yoichi Kaya proposed a simple yet widely applicable model for quantifying carbon dioxide emissions at the 1989 IPCC conference. The "Kaya Identity" model links economic, social, and environmental factors to calculate the carbon dioxide generated from human activities. It incorporates various important factors that influence carbon dioxide emissions and accurately.

Definitions of each variable are shown in Table 1 below:

**Table 1:** Explanation of variables in the Kaya Identity.

Variable	Significance	Unit
P	Population	10,000 people
GDP	Regional Gross Domestic Product(GDP)	100 billion CNY
E	Energy consumption	10,000 metric tonnes of standard coal
$\frac{GDP}{P}$	Per capita GDP	10,000 CNY/person
$\frac{E}{GDP}$	Energy consumption per unit of GDP	metric tonnes of coal equivalent per 10,000 CNY
$\frac{C}{E}$	Carbon dioxide emissions per unit of energy consumption	metric tonnes per 10,000 CNY

By combining relevant variables and incorporating new values based on the selected range of influencing factors, the Kaya identity can be expressed mathematically as shown in Eq 1. Additionally, Eq 2 represents a simplified form that reflects how influencing factors affect changes in carbon emissions from public buildings. This equation serves as a fundamental tool for computing historical carbon dioxide emission levels of public buildings in Fujian Province.


$$C=P\cdot \frac{GDP}{P}\cdot \frac{G_{t}}{GDP}\cdot \frac{F_{c}}{G_{t}}\cdot \frac{E}{F_{c}}\cdot \frac{C}{E}$$ (1)

$$C=P\cdot G\cdot R\cdot F\cdot EF\cdot C_{c}$$ (2)

The specific variables are as shown in Table 2.

**Table 2:** Specific variable settings.

Variable	Significance	Unit
*F*_*c*_	Total area of public buildings	10,000 square meters
*G*_*t*_	Gross Domestic Product (GDP) of the tertiary sector	10,000 CNY
$G=\frac{GDP}{P}$	Regional per capita GDP	CNY
$R=\frac{G_{t}}{GDP}$	Percentage of the tertiary sector	%
$F=\frac{F_{c}}{G_{t}}$	Economic activity intensity of public buildings	metric tonnes of coal equivalent per 10,000 CNY
$EF=\frac{E}{F_{c}}$	Energy consumption per unit area of public buildings	10,000 metric tonnes of CO2
$C_{c}=\frac{C}{E}$	Total amount of carbon dioxide emissions per unit of energy consumption	10,000 metric tonnes of standard coal

#### STIRPAT model

The Kaya Identity and STIRPAT model both belong to the IPAT family, and they are classical methods used in macroscopic analysis to explore the influencing factors of historical changes in carbon emissions across sectors and conduct scenario analyses for future carbon emission peaking. STIRPAT is an extension of the IPAT model. The IPAT equation measures the interaction mechanisms among environmental impact, population development, wealth level, and scientific and technological progress to study the main causes of environmental impact. It is believed that population, wealth, and technology are the primary reasons influencing the environment. Therefore, this identity has been widely applied in research on the relationships between environment, population, economy, and technology [35]. The expression of the IPAT identity is as follows:

I=P×A×T (3)

Among them, I represents the environmental impact within the region, P represents population development, A represents wealth level, and T represents the scientific and technological level of cities. However, due to the significant limitations of this identity, subsequent scholars have also made improvements to the IPAT model. Dietz et al. proposed the STIRPAT model based on the IPAT model. The general expression is:

$$I=\alpha P^{b}A^{c}T^{d}e$$ (4)

Among them, α represents the coefficient of the model, b, c, and d represent the exponents of respective variables, and e represents the error. Taking the natural logarithm on both sides of Eq (4), we obtain the following equation:

lnI=lnα+blnP+clnA+dlnT+lne (5)

The equation of the STIRPAT model is a derivative form of the IPAT identity. When a, b, c, d, and e are all equal to 1, the equation becomes the IPAT identity.

The STIRPAT model is a non-linear model. Since it is impossible for the influencing factors and the environment to have proportional changes, the STIRPAT model addresses the limitation of IPAT model in effectively identifying the degree of influence of various factors on carbon dioxide emissions. After taking the logarithm of the formula of the model, the coefficient term in the formula can represent the elasticity relationship between variables. Since then, many studies have started using this model. Based on the STIRPAT model, the study uses linear regression analysis to estimate the impact of various influencing factors on changes in carbon emissions in public buildings.

From the definitions of the Kaya identity and the STIRPAT model, it can be seen that both are branches of the IPAT model. The STIRPAT model is more suitable for linear regression analysis. Specifically, this section converts the Kaya identity shown in Eq (2) into the STIRPAT model for easy calculation, as shown in Eq (6).


$$lnC=ln\alpha +\beta_{1}lnP+\beta_{2}lnG+\beta_{3}lnR+\beta_{4}lnF+\beta_{5}lnEF+\beta_{6}lnC_{c}+lne$$ (6)

#### Scenario analysis

Scenario Analysis, also referred to as a foresight technique, is a forecasting method employed to predict potential situations or outcomes by extrapolating the continuation of a phenomenon or trend into the future. It serves as an intuitive qualitative approach commonly utilized to make assumptions or predictions regarding the future development of the subject under consideration. By speculating on each influencing factor, this method classifies and describes plausible future scenarios, engaging in comprehensive discussions about them. Moreover, it integrates and summarizes factors exhibiting strong correlations to forecast the overall impacts. The efficacy of Scenario Analysis in forecasting future trends in carbon emissions has been substantiated.

### Data sources and processing

#### Data sources

The macro-level data on carbon emissions from public buildings during the operational stage, used in the study, were obtained from the Building Carbon Emission Statistical System developed by the Energy Consumption Statistics Committee of the China Association of Building Energy Efficiency. The research selected the relevant data on carbon emissions from urban public buildings in Fujian from 2010 to 2020. In addition, the six characteristic indicators of influencing factors mentioned in the study- total population, per capita GDP, the proportion of the tertiary industry, economic activity intensity of public buildings, per unit of floor space consumption, and carbon dioxide emissions per unit of energy consumption ‐ were all obtained from the "Fujian Statistical Yearbook" (2010–2020). To conform to the requirements of the STIRPAT model, the data were logarithmically transformed, and the results of the data processing are shown in Table 3.

**Table 3:** Data processing values of each influencing factor.

Year	*lnC*	*lnP*	*lnG*	*lnR*	*lnF*	*lnEF*	*lnC*_*c*_
2010	7.335159293	8.214194415	10.61211876	-0.911903439	1.033973221	-0.61223448	-1.790648808
2011	7.531529337	8.23853693	10.76534777	-0.924434498	0.972529444	-0.633277861	-1.676832078
2012	7.603964766	8.253488028	10.86983121	-0.921442725	0.974852028	-0.7356042	-1.626819204
2013	7.658314397	8.264878263	10.96690335	-0.921678302	0.96263893	-0.766020507	-1.638066965
2014	7.643072665	8.280204233	11.05444735	-0.921887094	0.968027868	-0.795074411	-1.732304913
2015	7.640031822	8.290041619	11.11718178	-0.877603944	1.002872755	-0.940989944	-1.741130067
2016	7.684957262	8.298041661	11.20814688	-0.840163713	0.917832349	-0.9778602	-1.710699345
2017	7.717014767	8.310169022	11.32964227	-0.791427866	0.812742452	-1.012931938	-1.720838796
2018	7.732695342	8.319717387	11.45390179	-0.795553707	0.763263831	-1.048257045	-1.750036546
2019	7.728765631	8.327726166	11.53578474	-0.766545867	0.689522366	-1.04966032	-1.797721085
2020	7.732459968	8.333510709	11.55983817	-0.744669874	0.696621216	-1.094942422	-1.807557463

#### Scenario settings for forecasting

We understand that there are numerous emission scenarios, such as beyond shared socio-economic pathways (SSPs), representative concentration pathways (RCPs), regional representative agricultural pathways (RAPs), and others. However, based on the current situation in Fujian Province, we have devised more suitable scenarios. This study aims to project the peak of carbon emissions from public buildings in Fujian Province through the construction of three distinct scenarios: the low-carbon scenario, baseline scenario, and high-carbon scenario. The baseline scenario forecasts the future trajectory of carbon emissions from public buildings based on current factors such as population, regional GDP, public building area, energy consumption levels, energy-saving measures, emission reduction standards, and provincial policies. In contrast, the low-carbon scenario introduces additional energy-saving strategies and emission reduction measures to expedite the attainment of the peak carbon emissions from public buildings. Conversely, the high-carbon scenario considers limitations or obstacles that impede the implementation of existing energy-saving measures, emission reduction standards, and policies outlined in the baseline scenario, thereby resulting in a delayed achievement of the peak carbon emissions from public buildings.

## Analysis and results

### Analysis of influencing factors

Before determining which regression model to use, it is necessary to evaluate the multicollinearity among variables. In the study, the data in the model were initially subjected to multiple linear regression using the least squares method, and the regression results obtained are shown in Table 4.

**Table 4:** Regression results using the least squares method.

Results of Linear Regression Analysis. n = 11									
	Non-normalized coefficients		Normalization factor	t	P	VIF	R2	Adjust R²	F
	B	standard error	Beta						
Constant	-9.21	0	-	-1153703683.749	0.000***	-	1	1	F = 1.2758500132252809e+21 P = 0.000***
*lnP*	1	0	0.326	729058206.494	0.000***	1531.529			
*lnG*	1	0	2.682	2811704793.308	0.000***	6965.529			
*lnR*	1	0	0.597	2178607432.276	0.000***	575.585			
*lnF*	1	0	1.069	2739839347.154	0.000***	1166.393			
*lnEF*	1	0	1.487	3497060098.872	0.000***	1384.272			
*lnC*_*c*_	1	0	0.516	10119773067.292	0.000***	19.901			
Dependent variable:Total carbon emissions from public buildings (10,000 metric tonnes of CO_2_)									

Note: ***, **, and * represent significance levels of 1%, 5%, and 10%, respectively.

The variables in Table 5 were preliminarily evaluated as follows: The F-test result indicates a highly significant level of P value (0.000***), thereby rejecting the null hypothesis that the regression coefficient is 0, and concluding that the model satisfies the basic requirements. Notably, the total population, per capita GDP, proportion of the tertiary industry, intensity of economic activity in public buildings, energy consumption per unit area of public buildings, and total CO_2_ emissions generated by unit energy consumption all have a substantial positive impact on the total carbon emissions of public buildings.

**Table 5:** Ridge regression analysis results.

K = 0.161	Non-normalized coefficients		Normalization factor	t	P	R^2^	Adjust R^2^	F
	B	standard error	Beta					
Constant	-4.154	1.726	-	-2.407	0.074*	0.966	0.916	19.118(0.007***)
Total population (in hundred million people)	1.279	0.186	0.417	6.865	0.002***			
Per capita GDP	0.125	0.016	0.335	7.574	0.002***			
Proportion of the tertiary industry	-0.135	0.141	-0.081	-0.959	0.392			
Intensity of economic activity in public buildings	-0.093	0.087	-0.099	-1.069	0.345			
Energy consumption per unit CO_2_ area of public buildings	-0.164	0.046	-0.244	-3.536	0.024**			
Total amount of emissions generated by unit energy consumption	0.707	0.169	-0.365	4.175	0.014**			
Total amount of emissions generated by unit energy consumption								

Note: ***, **, and * represent significance levels of 1%, 5%, and 10%, respectively.

**Fig 1:**
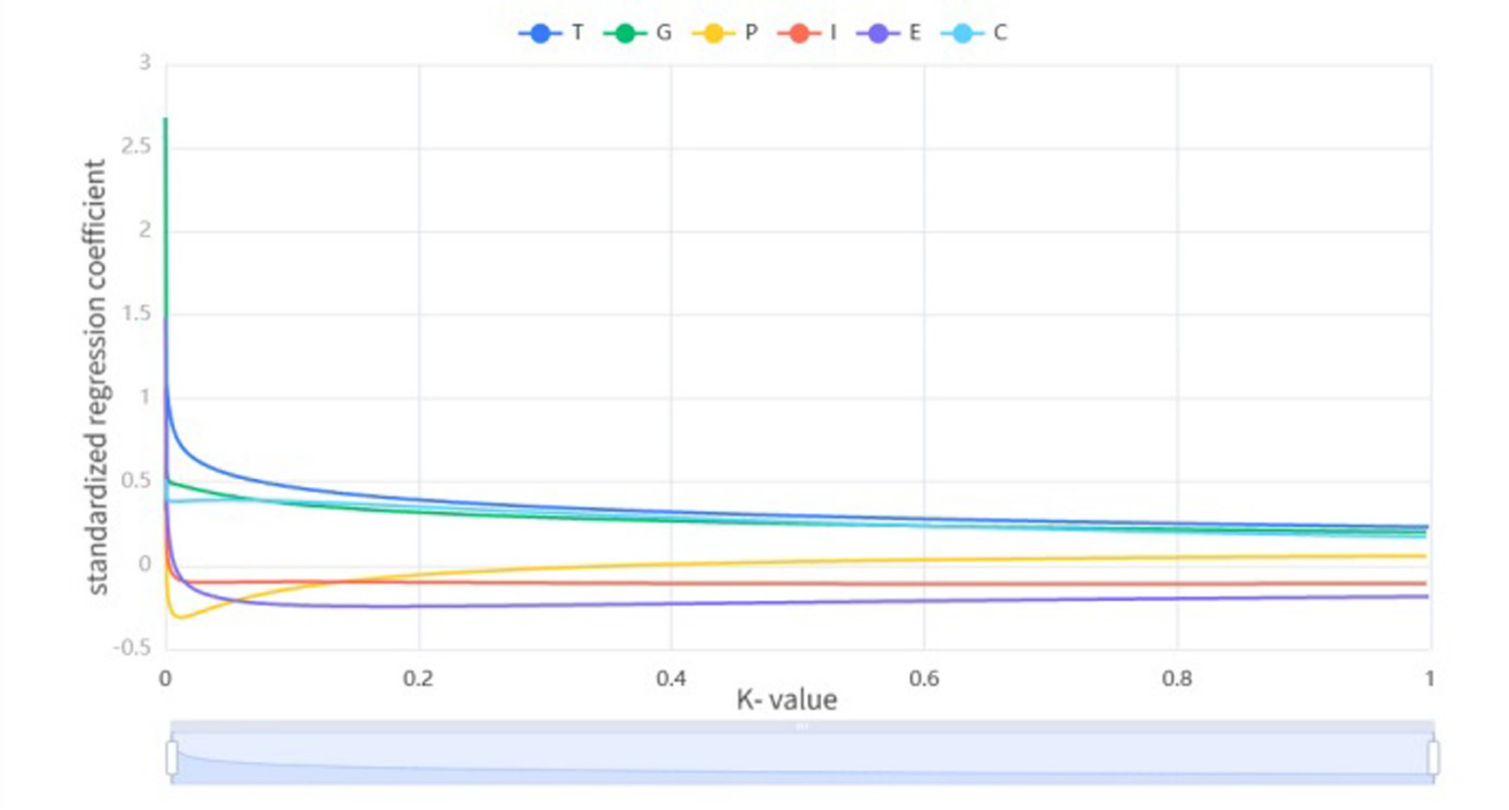
Ridge trace plot. T (Total population in hundreds of millions), G (Per capita GDP), P (Proportion of the tertiary industry), I (Intensity of economic activity in public buildings), E (Energy consumption per unit area of public buildings), and C (Total amount of CO2 emissions generated by unit energy consumption).

Collinearity among variables was observed, with VIF values exceeding 10 for each independent variable. To alleviate collinearity, we suggest removing the collinear independent variables or adopting techniques such as ridge regression or stepwise regression (Fig 1).

In order to solve the collinearity problem among variables, the paper uses Ridge Regression [36] to re-analyze the regression. Ridge Regression analysis is used to solve the algorithm for independent variable collinearity in linear regression analysis.

The paper uses SPSSPRO software [37] to perform Ridge Regression analysis on the influencing factors. According to the trend of the ridge trace plot and the variance expansion factor method, K is determined to be 0.161, and the results are shown in Table 5.

The F test found a highly significant P value of 0.007***, thereby rejecting the null hypothesis and indicating a regression relationship between the independent variables and the dependent variable. The model’s goodness of fit R² is 0.966, signifying excellent model performance. Regression coefficients portray positive associations between the total population, per capita GDP, and total amount of CO_2_ emissions generated by unit energy consumption with carbon emissions from public buildings. Conversely, negative correlations exist between the proportion of tertiary industries, energy consumption per unit area of public buildings, and intensity of economic activity in public buildings with carbon emissions from public buildings. Specifically, a 1% rise in Fujian Province’s population contributes to a 1.279% increase in carbon emissions from public buildings, while a 1% elevation in per capita GDP leads to a 0.125% increase. Conversely, a 1% growth in the proportion of the tertiary industry results in a 0.135% reduction in CO_2_ emissions from public buildings. A 1% increase in the intensity of economic activity in public buildings and energy consumption per unit area of public buildings corresponds to a 0.093 and 0.164 decrease, respectively, in carbon emissions from public buildings. A 1% increase in the total amount of CO_2_ emissions generated by unit energy consumption increases carbon emissions from public buildings by 0.707%. Eq 7 presents the ultimate STIRPAT model formula.


$$\begin{array}{c} lnC=-4.154+1.279lnP+0.125lnG-0.135lnR-0.093lnF-\\ 0.164lnEF+0.707lnC_{c} \end{array}$$ (7)

### Carbon emission forecasting

**Fig 2:**
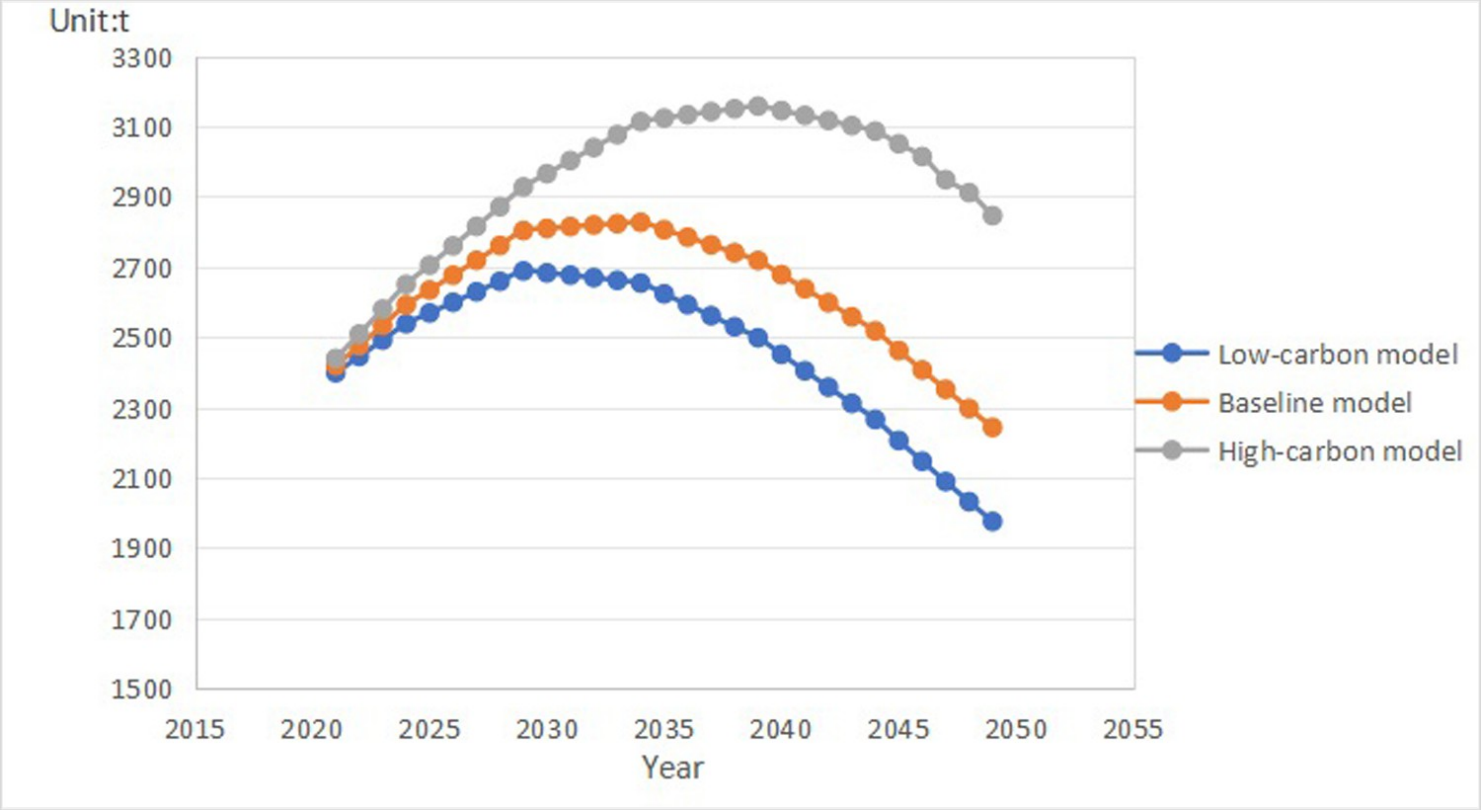
Carbon peaking data in each scenario. The blue line represents Low-corbon model, orange line represents Baseline model, gray line represents High-carbon model.

Using the established STIRPAT model, we inputted specific parameters of various influencing factors in scenario predictions to determine the total carbon emissions and carbon peak time of public buildings in Fujian Province from 2021 to 2050 under low-carbon, baseline, and high-carbon scenarios. Detailed values and trends can be found in Table 6 and Fig 2.

**Table 6:** Carbon peaks calculated in three context modes.

Year	Baseline scenario (t)	Low-carbon scenario(t)	High-carbon scenario(t)
2021	2362.172	2352.35	2372.05
2022	2418.668	2398.592	2439.439
2023	2475.668	2444.899	2508.657
2024	2533.433	2491.525	2579.782
2025	2591.656	2538.159	2650.132
2026	2633.836	2568.671	2704.603
2027	2676.087	2598.946	2759.565
2028	2718.39	2628.967	2815
2029	2760.724	2658.715	2870.893
2030	2803.069	2688.171	2927.224
2031	2809.243	2682.7	2965.048
2032	2814.45	2676.298	3002.325
2033	2819.011	2669.284	3039.369
2034	2822.92	2661.662	3076.159
2035	2826.505	2653.748	3113.035
2036	2805.673	2623.071	3123.215
2037	2784.342	2592.134	3132.7
2038	2762.525	2560.956	3141.485
2039	2740.234	2529.552	3149.559
2040	2717.803	2498.236	3157.286
2041	2677.841	2451.049	3144.389
2042	2637.843	2404.18	3130.811
2043	2597.827	2357.645	3116.562
2044	2557.813	2311.457	3101.649
2045	2517.817	2265.631	3086.082
2046	2461.782	2205.74	3050.104
2047	2406.42	2146.915	3013.833
2048	2351.46	2088.903	2948.05
2049	2296.93	2031.722	2910.922
2050	2243.125	1975.626	2845.695

The Fig 2 and Table 6 indicate that the carbon emissions of public buildings in Fujian Province will reach their peak in 2030, 2035, and 2040 under the low-carbon, baseline, and high-carbon scenarios, respectively. In the baseline scenario, carbon emissions show a year-on-year increase from 2021 to 2035, peaking in 2035, and decreasing thereafter. Consequently, it is advisable to reinforce energy-saving and emission-reduction measures beyond the current policies to attain national targets. The low-carbon scenario depicts a gradual increase in carbon emissions of public buildings from 2021 to 2030, with a peak in 2030, followed by a gradual decrease. In contrast, the high-carbon scenario’s public building carbon emissions peak in 2040 within the 2021–2040 period before gradually declining.

## Discussion

The figure and table indicate that the carbon emissions of public buildings in Fujian Province will reach their peak in 2030, 2035, and 2040 under the low-carbon, baseline, and high-carbon scenarios, respectively. In the baseline scenario, carbon emissions show a year-on-year increase from 2021 to 2035, peaking in 2035, and decreasing thereafter. Consequently, it is advisable to reinforce energy-saving and emission-reduction measures beyond the current policies to attain national targets. The low-carbon scenario depicts a gradual increase in carbon emissions of public buildings from 2021 to 2030, with a peak in 2030, followed by a gradual decrease. In contrast, the high-carbon scenario’s public building carbon emissions peak in 2040 within the 2021–2040 period before gradually declining.

### Baseline scenario

According to the predictions, carbon dioxide emissions from public buildings in Fujian Province are projected to exhibit an average annual growth rate of approximately 2.3% from 2021 to 2025. This growth rate is anticipated to decrease to around 1.6% between 2025 and 2030, and further decrease to a mere 0.16% from 2031 to 2035. The decline in the growth rate signifies the successful implementation of energy-saving and emission-reduction measures in the province. Although the baseline scenario estimates the carbon peak year to be 2035, which falls short of the national target of achieving carbon peak by 2030, the substantial reduction in the average annual growth rate of carbon emissions from public buildings between 2031 and 2035 implies that there is potential for further strengthening the energy-saving and emission-reduction policies. Possible measures include enhancing public awareness of energy-saving and emission-reduction practices, promoting the development of green and energy-saving technologies, and reducing primary energy consumption. These actions would enable the province to meet the national requirements for carbon peak and expedite its attainment.

### Low-carbon scenario

In comparison to the baseline scenario, the low-carbon scenario demonstrates a slower growth rate in population, economic development, and energy consumption. Under this low-carbon development scenario, the peak of carbon emissions from public buildings in Fujian Province is expected to occur earlier and at a relatively lower magnitude, thus aligning with the carbon peak target around 2030. The carbon peak value decreases by approximately 13.5% when compared to the baseline scenario, indicating that the improved implementation of carbon emission policies yields more effective energy-saving and emission-reduction outcomes. This suggests that within the low-carbon development scenario, the adjustment of industrial structure and reduction in energy consumption levels will expedite the decline of carbon emissions from public buildings. Consequently, it will facilitate the early attainment of the carbon emissions peak, reduce cumulative carbon emissions, and mitigate their environmental impact.

### High-carbon scenario

Under the high-carbon development scenario, the six factors influencing the carbon emissions of public buildings in Fujian Province exhibit high growth rates. As a consequence, the carbon peak value is anticipated to transpire around 2040, significantly surpassing China’s objective of attaining carbon peak before 2030. It can be computed that the carbon peak value of public buildings in the high-carbon scenario exceeds that of the baseline scenario by approximately 12.5%.

## Conclusions and policy implications

This study initially analyzes the current factors affecting carbon emissions from public buildings in Fujian Province. It subsequently employs the Kaya Identity, STIRPAT model, and ridge regression calculation methods to identify the factors influencing carbon emissions and generate a regression curve for the past decade. Moreover, three scenario models are constructed using scenario analysis, and parameters are set based on future development conditions to compare and analyze the carbon peak time and peak level under these models. Results indicate that the carbon peak time for public buildings in Fujian Province is predicted to occur in 2035 for the baseline model, 2030 for the low-carbon model, and 2040 for the high-carbon model. Only under the low-carbon model is it feasible for urban public buildings in Fujian Province to attain the carbon peak goal of 2030.

(1) Adhere to a high-quality development approach and maintain healthy economic growth

In order to meet the target of carbon peak in public buildings by 2030, upholding current development policies alone falls short. As a result, adhering to a high-quality economic development strategy not only becomes an essential requirement for maintaining sound economic growth but also holds the key to advancing the timing of the carbon peak in public buildings. Given that new low-carbon technologies are often complex and costly due to rapid advancements in social technology and continuous innovation, insufficient funding may lead to losses against traditional production models. Therefore, China should increase financial support for emerging technology industries such as through taxation and fiscal subsidies, gradually phasing out these supports as these industries mature.

(2) Accelerate industrial structure upgrading and increase the proportion of the tertiary industry

From the previous discussion, we can conclude that the tertiary industry has a restraining effect on carbon emissions from public buildings. The continuous upgrading of the industrial structure will shorten the time to achieve carbon peaking in public buildings. Industrial structure upgrading does not mean completely abandoning the primary and secondary industries, but rather refers to accelerating the adjustment of the industrial structure and increasing the proportion of the tertiary industry. We need to ensure the normal operation of advantageous industries and promote the innovative development of high-tech industries. By incorporating high-tech and low-carbon energy-saving equipment and replacing traditional machinery and equipment, we can reduce carbon emissions and adjust the internal structure of the secondary industry. Finally, we should vigorously promote the development of the tertiary industry. The tertiary industry is the focus of future economic development. With the continuous growth of the population, the demand for services will also increase, which in turn will drive the demand for public buildings. Therefore, by utilizing local abundant historical, cultural, and tourism resources and combining them with local conditions, we can create unique tourism and cultural characteristics to attract talent transfer to the tertiary industry, thus accelerating the optimization and upgrading of the industrial structure.

(3) Develop green building plans to reduce energy consumption level

Due to rapid economic and social development, the proportion of public buildings continues to rise. It is important to develop green building plans to achieve energy-saving and emission reduction. The government needs to increase the implementation efforts of green buildings and plan the overall goals for future development of the construction industry. While promoting green buildings and energy-saving measures, specific requirements should be proposed, emphasizing the construction of low-energy buildings and increasing the use of green and low-carbon building materials. Green upgrades should also be carried out for existing buildings that are suitable for renovation. Additionally, corresponding laws and regulations can be established, and efforts can be made to organize and coordinate the development of nearly zero-energy buildings.

Local governments should also actively conduct energy audits, investigate the energy consumption of public buildings in various areas, gain in-depth understanding of the energy consumption status, and compile statistical data. A complete evaluation and assessment system and statistical analysis methods should be established for subsequent adjustments and upgrades of policies related to low-energy consumption in public buildings [38, 39].

(4) Open the carbon trading market and strictly control carbon emissions

To control carbon emissions in building construction, we can start with the carbon trading market. The carbon trading market is an important area for energy conservation and emission reduction. Establishing a carbon trading market helps to enhance the economic value of green and low-carbon projects and technologies from a market-oriented perspective, promote the transformation of enterprises to green and low-carbon production methods, and has a significant role in promoting the achievement of carbon peak and carbon neutrality goals [40].

The government should actively play the role of the market economy, implement a carbon trading mechanism, and motivate energy-consuming units in buildings to participate in carbon emissions trading. Based on innovation to improve energy conservation and emission reduction technologies, benefits can be obtained and costs reduced through the carbon emissions trading market. Since public buildings are generally large buildings, reducing their energy consumption will have a significant impact. Finally, the government can also establish practical carbon tax policies to achieve the goal of reducing energy consumption, improving energy efficiency, and reducing carbon emissions intensity [41].

(5) Vigorously promote low-carbon policies and promote a nationwide low-carbon action

Although the fertility rate in China is gradually declining, the total population is still growing. As the main users of public buildings, improving the national low-carbon awareness is the foundation for achieving carbon peak and carbon neutrality as soon as possible. The government should actively carry out a nationwide low-carbon action, increase publicity efforts, raise people’s awareness of energy conservation and emission reduction, increase understanding of green buildings and their importance. For key energy conservation and emission reduction areas, local community organizations can be arranged to conduct training activities on this topic, so that people can develop low-carbon habits in their daily lives and work, such as turning off lights when leaving a room, using energy-saving appliances, etc. At the same time, the government can also set up a green energy-saving building demonstration area or hold a green building exhibition, to promote energy conservation and emission reduction in surrounding areas through demonstration actions.

## Supporting information

S1 Table Data related to carbon emissions from urban and rural public buildings in Fujian Province, 2010–2020. (PDF)

S2 Table Data on various factors influencing carbon emissions from urban and rural public buildings in Fujian Province, 2010–2020. (PDF)

S3 Table Data projections for each impact factor in the baseline model, 2021–2050. (PDF)

S4 Table Data projections for each influencing factor in the low-carbon model, 2021–2050. (PDF)

S5 Table Data projections for each influencing factor in the high-carbon model, 2021–2050. (PDF)

S6 Table Data processing values for each impact factor in the baseline model, 2021–2050. (PDF)

S7 Table Data processing values for each impact factor in the low-carbon model, 2021–2050. (PDF)

S8 Table Data processing values for each impact factor in the high-carbon model, 2021–2050. (PDF)
