# Supplementary material for: Prediction of carbon emissions from public buildings in China’s Coastal Provinces under different scenarios ——A case study of Fujian Province
Source: PLoS One. 2024 Jul 23;19(7):e0307201. doi: 10.1371/journal.pone.0307201 (PMC11265700; doi:10.1371/journal.pone.0307201)
Supplement: S1 Table — (PDF) [file pone.0307201.s001.pdf]

S1 Table. Data related to carbon emissions from urban and rural public buildings in Fujian Province, 2010-2020

| Year | Population (10,000 people) | GDP（10,000 CNY） | Gross domestic product of the tertiary sector（10,000 CNY） | Total area of public buildings（10,000 square meters） | Energy consumption(10,000 metric tonnes of standard coal) | Total carbon emissions from public buildings（10,000 metric tonnes of CO2） |
|---|---|---|---|---|---|---|
| 2010 | 3693 | 150025100 | 60273900 | 16950.33 | 9189.42 | 1533.272 |
| 2011 | 3784 | 179177000 | 71089500 | 18800.51 | 9980.23 | 1865.957 |
| 2012 | 3841 | 201907300 | 80347900 | 21298.42 | 10206.54 | 2006.134 |
| 2013 | 3885 | 225038400 | 89531700 | 23444.75 | 10898.51 | 2118.184 |
| 2014 | 3945 | 249420700 | 99211500 | 26119.88 | 11794.37 | 2086.144 |
| 2015 | 3984 | 268194600 | 111509400 | 30398.6 | 11862.79 | 2079.81 |
| 2016 | 4016 | 296094300 | 127806100 | 32000.82 | 12035.99 | 2175.377 |
| 2017 | 4065 | 338424400 | 153373000 | 34571.52 | 12554.74 | 2246.244 |
| 2018 | 4104 | 386877700 | 174610000 | 37458.5 | 13131.01 | 2281.744 |
| 2019 | 4137 | 423265800 | 196655700 | 39188.83 | 13718.31 | 2272.795 |
| 2020 | 4161 | 436085500 | 207093100 | 41562.76 | 13905.19 | 2281.207 |
