# Supplementary material for: Prediction of carbon emissions from public buildings in China’s Coastal Provinces under different scenarios ——A case study of Fujian Province
Source: PLoS One. 2024 Jul 23;19(7):e0307201. doi: 10.1371/journal.pone.0307201 (PMC11265700; doi:10.1371/journal.pone.0307201)
Supplement: S2 Table — (PDF) [file pone.0307201.s002.pdf]

S2 Table. Data on various factors influencing carbon emissions from urban and rural public buildings in Fujian Province, 2010-2020

Year	Population (10,000 people)	Regional per capita GDP(CNY)	Percentage of the tertiary sector	Economic activity intensity of public buildings	Energy consumption per unit area of public buildings	Total amount of carbon dioxide emissions per unit of energy consumption
2010	3693	40624.18088	0.401758772	0.000281222	0.542138118	0.16685188
2011	3784	47351.21564	0.396755722	0.000264463	0.530848897	0.18696533
2012	3841	52566.33689	0.397944502	0.000265077	0.479215829	0.196553778
2013	3885	57924.94208	0.397850767	0.00026186	0.464859297	0.194355375
2014	3945	63224.51204	0.397767707	0.000263275	0.451547633	0.176876255
2015	3984	67317.92169	0.415777946	0.00027261	0.390241327	0.175322163
2016	4016	73728.66036	0.431639853	0.000250386	0.37611505	0.180739349
2017	4065	83253.23493	0.453197228	0.000225408	0.363152676	0.178916011
2018	4104	94268.44542	0.45133126	0.000214527	0.350548207	0.173767593
2019	4137	102312.2553	0.464615143	0.000199276	0.350056636	0.16567602
2020	4161	104803.0522	0.474891048	0.000200696	0.33455887	0.164054357
