# Supplementary material for: Prediction of carbon emissions from public buildings in China’s Coastal Provinces under different scenarios ——A case study of Fujian Province
Source: PLoS One. 2024 Jul 23;19(7):e0307201. doi: 10.1371/journal.pone.0307201 (PMC11265700; doi:10.1371/journal.pone.0307201)
Supplement: S3 Table — (PDF) [file pone.0307201.s003.pdf]

S3 Table. Data projections for each impact factor in the baseline model, 2021-2050

Year	Population (10,000 people)	Regional per capita GDP(CNY)	Percentage of the tertiary sector	Economic activity intensity of public buildings	Energy consumption per unit area of public buildings	Total amount of carbon dioxide emissions per unit of energy consumption
2021	4202.61	115283.3574	0.482964196	0.000188654	0.324522103	0.16192165
2022	4244.6361	126581.1264	0.491174587	0.000177335	0.31478644	0.159816669
2023	4287.082461	138606.3334	0.499524555	0.000166695	0.305342847	0.157739052
2024	4329.953286	151496.7224	0.508016472	0.000156693	0.296182562	0.155688444
2025	4373.252818	165131.4274	0.516652752	0.000147292	0.287297085	0.153664494
2026	4395.119083	179662.993	0.525435849	0.000138454	0.278678172	0.151666856
2027	4417.094678	195114.0104	0.534368259	0.000130147	0.270317827	0.149695187
2028	4439.180151	211503.5873	0.543452519	0.000122338	0.262208292	0.147749149
2029	4461.376052	228846.8815	0.552691212	0.000114998	0.254342044	0.145828411
2030	4483.682932	247154.632	0.562086962	0.000108098	0.246711782	0.143932641
2031	4461.264518	266432.6933	0.571642441	0.000101612	0.239310429	0.142061517
2032	4438.958195	286415.1453	0.581360362	0.000095515	0.232131116	0.140214717
2033	4416.763404	307323.4509	0.591243488	0.000089784	0.225167182	0.138391926
2034	4394.679587	329143.4159	0.601294628	0.000084397	0.218412167	0.136592831
2035	4372.706189	352183.455	0.611516636	0.000079333	0.211859802	0.134817124
2036	4328.979127	376131.9299	0.617631803	0.000076953	0.205504008	0.133064501
2037	4285.689336	400956.6373	0.623808121	0.000074645	0.199338888	0.131334663
2038	4242.832443	426617.8621	0.630046202	0.000072406	0.193358721	0.129627312
2039	4200.404118	453068.1696	0.636346664	0.000070233	0.187557959	0.127942157
2040	4158.400077	480705.3279	0.642710131	0.000068126	0.181931221	0.126278909
2041	4096.024076	509547.6476	0.649137232	0.000066083	0.176473284	0.124637283
2042	4034.583715	539101.4111	0.655628604	0.000064100	0.171179085	0.123016999
2043	3974.064959	569291.0902	0.66218489	0.000062177	0.166043713	0.121417778
2044	3914.453985	600032.809	0.668806739	0.000060312	0.161062402	0.119839347
2045	3855.737175	631234.5151	0.675494807	0.000058502	0.156230529	0.118281435
2046	3778.622431	662796.2408	0.682249755	0.000056747	0.151543614	0.116743776
2047	3703.049983	694610.4604	0.689072252	0.000055045	0.146997305	0.115226107
2048	3628.988983	725867.9311	0.695962975	0.000053394	0.142587386	0.113728168
2049	3556.409203	756354.3842	0.702922604	0.000051792	0.138309764	0.112249702
2050	3485.281019	786608.5596	0.70995183	0.000050238	0.134160472	0.110790456
