# Supplementary material for: Prediction of carbon emissions from public buildings in China’s Coastal Provinces under different scenarios ——A case study of Fujian Province
Source: PLoS One. 2024 Jul 23;19(7):e0307201. doi: 10.1371/journal.pone.0307201 (PMC11265700; doi:10.1371/journal.pone.0307201)
Supplement: S4 Table — (PDF) [file pone.0307201.s004.pdf]

S4 Table. Data projections for each influencing factor in the low-carbon model, 2021-2050

Year	Population (10,000 people)	Regional per capita GDP(CNY)	Percentage of the tertiary sector	Economic activity intensity of public buildings	Energy consumption per unit area of public buildings	Total amount of carbon dioxide emissions per unit of energy consumption
2021	4192.2075	114235.3268	0.48058974	0.000190661	0.327867692	0.162413813
2022	4223.649056	124288.0356	0.486356817	0.000181128	0.321310338	0.160789675
2023	4255.326424	134852.5186	0.492193099	0.000172072	0.314884132	0.159181778
2024	4287.241372	146045.2777	0.498099416	0.000163468	0.308586449	0.15758996
2025	4319.395683	157728.8999	0.504076609	0.000155295	0.30241472	0.156014061
2026	4330.194172	170031.7541	0.510125528	0.000147530	0.296366426	0.15445392
2027	4341.019657	182954.1674	0.516247035	0.000140154	0.290439097	0.152909381
2028	4351.872206	196492.7758	0.522441999	0.000133146	0.284630315	0.151380287
2029	4362.751887	210640.2556	0.528711303	0.000126489	0.278937709	0.149866484
2030	4373.658767	225385.0735	0.535055839	0.000120164	0.273358955	0.14836782
2031	4340.856326	240711.2585	0.541476509	0.000114156	0.267891776	0.146884141
2032	4308.299903	256357.4903	0.547974227	0.000108448	0.26253394	0.1454153
2033	4275.987654	272508.0122	0.554549918	0.000103026	0.257283261	0.143961147
2034	4243.917747	289131.001	0.561204517	0.000097874	0.252137596	0.142521535
2035	4212.088364	306478.861	0.567938971	0.000092981	0.247094844	0.14109632
2036	4159.437259	324254.635	0.570778666	0.000091121	0.242152947	0.139685357
2037	4107.444293	342412.8945	0.573632559	0.000089299	0.237309888	0.138288503
2038	4056.10124	360903.1908	0.576500722	0.000087513	0.23256369	0.136905618
2039	4005.399974	379670.1568	0.579383226	0.000085762	0.227912417	0.135536562
2040	3955.332475	399033.3348	0.582280142	0.000084047	0.223354168	0.134181196
2041	3886.114156	418985.0015	0.585191542	0.000082366	0.218887085	0.132839385
2042	3818.107159	439096.2816	0.5881175	0.000080719	0.214509343	0.131510991
2043	3751.290283	459294.7105	0.591058088	0.000079105	0.210219156	0.130195881
2044	3685.642703	479503.6778	0.594013378	0.000077522	0.206014773	0.128893922
2045	3621.143956	499642.8323	0.596983445	0.000075972	0.201894478	0.127604983
2046	3539.668217	519628.5455	0.599968362	0.000074453	0.197856588	0.126328933
2047	3460.025682	539374.4303	0.602968204	0.000072964	0.193899456	0.125065644
2048	3382.175104	558252.5353	0.605983045	0.000071504	0.190021467	0.123814987
2049	3306.076164	576116.6165	0.60901296	0.000070074	0.186221038	0.122576837
2050	3231.689451	593400.115	0.612058025	0.000068673	0.182496617	0.121351069
