# Supplementary material for: Prediction of carbon emissions from public buildings in China’s Coastal Provinces under different scenarios ——A case study of Fujian Province
Source: PLoS One. 2024 Jul 23;19(7):e0307201. doi: 10.1371/journal.pone.0307201 (PMC11265700; doi:10.1371/journal.pone.0307201)
Supplement: S5 Table — (PDF) [file pone.0307201.s005.pdf]

S5 Table. Data projections for each influencing factor in the high-carbon model, 2021-2050

Year	Population (10,000 people)	Regional per capita GDP(CNY)	Percentage of the tertiary sector	Economic activity intensity of public buildings	Energy consumption per unit area of public buildings	Total amount of carbon dioxide emissions per unit of energy consumption
2021	4213.0125	116331.3879	0.485338651	0.000186647	0.321176515	0.161429487
2022	4265.675156	129104.5743	0.496016101	0.000173582	0.308329454	0.158846615
2023	4318.996096	143241.5252	0.506928455	0.000161431	0.295996276	0.156305069
2024	4372.983547	158897.8239	0.518080881	0.000150131	0.284156425	0.153804188
2025	4427.645841	174787.6062	0.529478661	0.000139622	0.272790168	0.151343321
2026	4460.853185	191916.7917	0.541127191	0.000129848	0.261878561	0.148921828
2027	4494.309584	210340.8037	0.553031989	0.000120759	0.251403419	0.146539079
2028	4528.016906	230112.8392	0.565198693	0.000112306	0.241347282	0.144194454
2029	4561.977033	251283.2204	0.577633065	0.000104444	0.231693391	0.141887342
2030	4596.19186	273898.7102	0.590340992	0.000097133	0.222425655	0.139617145
2031	4607.68234	298001.7967	0.603328494	0.000090334	0.213528629	0.137383271
2032	4619.201546	323331.9495	0.616601721	0.000084011	0.204987484	0.135185138
2033	4630.74955	350168.5013	0.630166958	0.000078130	0.196787984	0.133022176
2034	4642.326424	378532.1499	0.644030632	0.000072661	0.188916465	0.130893821
2035	4653.93224	408814.7219	0.658199305	0.000067575	0.181359806	0.12879952
2036	4642.297409	440702.2702	0.668072295	0.000064872	0.174105414	0.126738728
2037	4630.691666	474195.6427	0.678093379	0.000062277	0.167141198	0.124710908
2038	4619.114936	509286.1203	0.68826478	0.000059786	0.16045555	0.122715534
2039	4607.567149	545954.7209	0.698588752	0.000057394	0.154037328	0.120752085
2040	4596.048231	584717.5061	0.709067583	0.000055098	0.147875835	0.118820052
2041	4561.577869	625647.7315	0.719703597	0.000052894	0.141960801	0.116918931
2042	4527.366035	668191.7773	0.730499151	0.000050779	0.136282369	0.115048228
2043	4493.41079	712292.4346	0.741456638	0.000048748	0.130831074	0.113207456
2044	4459.710209	757879.1504	0.752578488	0.000046798	0.125597831	0.111396137
2045	4426.262383	804867.6577	0.763867165	0.000044926	0.120573918	0.109613799
2046	4370.934103	853159.7172	0.775325172	0.000043129	0.115750961	0.107859978
2047	4316.297427	902642.9808	0.78695505	0.000041404	0.111120923	0.106134218
2048	4229.971478	952288.3447	0.798759376	0.000039747	0.106676086	0.104436071
2049	4177.096835	1001807.339	0.810740766	0.000038158	0.102409043	0.102765094
2050	4093.554898	1051897.706	0.822901878	0.000036631	0.098312681	0.101120852
