# Supplementary material for: Prediction of carbon emissions from public buildings in China’s Coastal Provinces under different scenarios ——A case study of Fujian Province
Source: PLoS One. 2024 Jul 23;19(7):e0307201. doi: 10.1371/journal.pone.0307201 (PMC11265700; doi:10.1371/journal.pone.0307201)
Supplement: S6 Table — (PDF) [file pone.0307201.s006.pdf]

S6 Table. Data processing values for each impact factor in the baseline model, 2021-2050

Year	Processed value of population (10,000 people)	Processed value of regional per capita GDP	Processed value of percentage of the tertiary sector	Processed value of economic activity intensity of public buildings	Processed value of energy consumption per unit area of public buildings	Processed value of total amount of carbon dioxide emissions per unit of energy consumption
2021	8.34346104	11.65514835	-0.727812757	-8.575594559	-1.12540163	-1.820642702
2022	8.353411371	11.7486387	-0.71095564	-8.637469963	-1.155860837	-1.833727942
2023	8.363361702	11.83939306	-0.694098523	-8.699345367	-1.186320045	-1.846813182
2024	8.373312032	11.92831927	-0.677241406	-8.76122077	-1.216779252	-1.859898421
2025	8.383262363	12.01449697	-0.660384289	-8.823096174	-1.24723846	-1.872983661
2026	8.388249905	12.09883811	-0.643527172	-8.884971578	-1.277697667	-1.8860689
2027	8.393237446	12.18133934	-0.626670055	-8.946846982	-1.308156875	-1.89915414
2028	8.398224988	12.26199724	-0.609812938	-9.008722385	-1.338616082	-1.912239379
2029	8.403212529	12.34080842	-0.592955821	-9.070597789	-1.36907529	-1.925324619
2030	8.408200071	12.41776946	-0.576098704	-9.132473193	-1.399534497	-1.938409858
2031	8.403187529	12.49287693	-0.559241587	-9.194348596	-1.429993705	-1.951495098
2032	8.398174987	12.56519759	-0.54238447	-9.256224	-1.460452912	-1.964580337
2033	8.393162445	12.63565606	-0.525527353	-9.318099404	-1.49091212	-1.977665577
2034	8.388149904	12.70424885	-0.508670236	-9.379974808	-1.521371327	-1.990750817
2035	8.383137362	12.7719075	-0.491813118	-9.441850211	-1.551830535	-2.003836056
2036	8.373087026	12.83769524	-0.481862788	-9.472309419	-1.582289742	-2.016921296
2037	8.36303669	12.90160856	-0.471912457	-9.502768626	-1.61274895	-2.030006535
2038	8.352986354	12.96364395	-0.461962126	-9.533227834	-1.643208157	-2.043091775
2039	8.342936018	13.02379788	-0.452011795	-9.563687041	-1.673667365	-2.056177014
2040	8.332885682	13.08300974	-0.442061464	-9.594146249	-1.704126572	-2.069262254
2041	8.317772045	13.14127865	-0.432111133	-9.624605456	-1.734585779	-2.082347493
2042	8.302658407	13.19765898	-0.422160802	-9.655064664	-1.765044987	-2.095432733
2043	8.287544769	13.25214716	-0.412210472	-9.685523871	-1.795504194	-2.108517973
2044	8.272431131	13.30473961	-0.402260141	-9.715983079	-1.825963402	-2.121603212
2045	8.257317493	13.35543273	-0.39230981	-9.746442286	-1.856422609	-2.134688452
2046	8.237114786	13.40422289	-0.382359479	-9.776901494	-1.886881817	-2.147773691
2047	8.216912079	13.45110648	-0.372409148	-9.807360701	-1.917341024	-2.160858931
2048	8.196709371	13.49512336	-0.362458817	-9.837819909	-1.947800232	-2.17394417
2049	8.176506664	13.53626531	-0.352508486	-9.868279116	-1.978259439	-2.18702941
2050	8.156303957	13.57548602	-0.342558156	-9.898738324	-2.008718647	-2.200114649
