# Supplementary material for: Prediction of carbon emissions from public buildings in China’s Coastal Provinces under different scenarios ——A case study of Fujian Province
Source: PLoS One. 2024 Jul 23;19(7):e0307201. doi: 10.1371/journal.pone.0307201 (PMC11265700; doi:10.1371/journal.pone.0307201)
Supplement: S7 Table — (PDF) [file pone.0307201.s007.pdf]

S7 Table. Data processing values for each impact factor in the low-carbon model, 2021-2050

Year	Processed value of population (10,000 people)	Processed value of regional per capita GDP	Processed value of percentage of the tertiary sector	Processed value of economic activity intensity of public buildings	Processed value of energy consumption per unit area of public buildings	Processed value of total amount of carbon dioxide emissions per unit of energy consumption
2021	8.340982724	11.64601587	-0.732741304	-8.56501245	-1.11514513	-1.817607799
2022	8.348454739	11.73035702	-0.720812733	-8.616305744	-1.135347837	-1.827658135
2023	8.355926753	11.81193701	-0.708884162	-8.667599039	-1.155550544	-1.83770847
2024	8.363398768	11.89167197	-0.696955591	-8.718892333	-1.175753252	-1.847758806
2025	8.370870783	11.96863301	-0.68502702	-8.770185627	-1.195955959	-1.857809142
2026	8.373367663	12.04374049	-0.673098449	-8.821478922	-1.216158666	-1.867859478
2027	8.375864544	12.11699095	-0.661169878	-8.872772216	-1.236361374	-1.877909814
2028	8.378361424	12.18838095	-0.649241308	-8.924065511	-1.256564081	-1.88796015
2029	8.380858304	12.25790701	-0.637312737	-8.975358805	-1.276766788	-1.898010486
2030	8.383355184	12.32556566	-0.625384166	-9.026652099	-1.296969495	-1.908060821
2031	8.375826918	12.3913534	-0.613455595	-9.077945394	-1.317172203	-1.918111157
2032	8.368298651	12.4543282	-0.601527024	-9.129238688	-1.33737491	-1.928161493
2033	8.360770385	12.5154233	-0.589598453	-9.180531983	-1.357577617	-1.938211829
2034	8.353242118	12.57463515	-0.577669882	-9.231825277	-1.377780325	-1.948262165
2035	8.345713852	12.63290406	-0.565741311	-9.283118571	-1.397983032	-1.958312501
2036	8.33313507	12.6892844	-0.56075377	-9.303321279	-1.418185739	-1.968362837
2037	8.320556288	12.74377258	-0.555766228	-9.323523986	-1.438388447	-1.978413172
2038	8.307977505	12.79636503	-0.550778687	-9.343726693	-1.458591154	-1.988463508
2039	8.295398723	12.84705815	-0.545791145	-9.363929401	-1.478793861	-1.998513844
2040	8.282819941	12.89680024	-0.540803604	-9.384132108	-1.498996569	-2.00856418
2041	8.265165006	12.9455904	-0.535816062	-9.404334815	-1.519199276	-2.018614516
2042	8.247510071	12.99247399	-0.530828521	-9.424537523	-1.539401983	-2.028664852
2043	8.229855135	13.03744735	-0.525840979	-9.44474023	-1.559604691	-2.038715188
2044	8.2122002	13.08050684	-0.520853438	-9.464942937	-1.579807398	-2.048765523
2045	8.194545265	13.12164879	-0.515865896	-9.485145645	-1.600010105	-2.058815859
2046	8.171788278	13.1608695	-0.510878355	-9.505348352	-1.620212813	-2.068866195
2047	8.149031291	13.19816528	-0.505890813	-9.525551059	-1.64041552	-2.078916531
2048	8.126274303	13.23256671	-0.500903272	-9.545753766	-1.660618227	-2.088966867
2049	8.103517316	13.26406538	-0.49591573	-9.565956474	-1.680820935	-2.099017203
2050	8.080760329	13.29362418	-0.490928189	-9.586159181	-1.701023642	-2.109067538
