# Supplementary material for: Prediction of carbon emissions from public buildings in China’s Coastal Provinces under different scenarios ——A case study of Fujian Province
Source: PLoS One. 2024 Jul 23;19(7):e0307201. doi: 10.1371/journal.pone.0307201 (PMC11265700; doi:10.1371/journal.pone.0307201)
Supplement: S8 Table — (PDF) [file pone.0307201.s008.pdf]

S8 Table. Data processing values for each impact factor in the high-carbon model, 2021-2050

Year	Processed value of population (10,000 people)	Processed value of regional per capita GDP	Processed value of percentage of the tertiary sector	Processed value of economic activity intensity of public buildings	Processed value of energy consumption per unit area of public buildings	Processed value of total amount of carbon dioxide emissions per unit of energy consumption
2021	8.345933229	11.66419819	-0.722908383	-8.586289848	-1.135764417	-1.823686845
2022	8.358355749	11.76837801	-0.701146891	-8.658860541	-1.176586411	-1.839816227
2023	8.370778269	11.87228747	-0.679385399	-8.731431234	-1.217408406	-1.855945609
2024	8.383200789	11.97601666	-0.657623907	-8.804001927	-1.2582304	-1.872074991
2025	8.395623309	12.07132684	-0.635862416	-8.87657262	-1.299052395	-1.888204373
2026	8.403095324	12.16481718	-0.614100924	-8.949143313	-1.339874389	-1.904333754
2027	8.410567339	12.25648437	-0.592339432	-9.021714005	-1.380696384	-1.920463136
2028	8.418039353	12.34632507	-0.57057794	-9.094284698	-1.421518378	-1.936592518
2029	8.425511368	12.43433595	-0.548816448	-9.166855391	-1.462340373	-1.9527219
2030	8.432983383	12.52051365	-0.527054957	-9.239426084	-1.503162367	-1.968851282
2031	8.435480263	12.60485479	-0.505293465	-9.311996777	-1.543984362	-1.984980664
2032	8.437977144	12.68643478	-0.483531973	-9.38456747	-1.584806357	-2.001110046
2033	8.440474024	12.76616975	-0.461770481	-9.457138162	-1.625628351	-2.017239428
2034	8.442970904	12.84405629	-0.440008989	-9.529708855	-1.666450346	-2.03336881
2035	8.445467784	12.92101733	-0.418247498	-9.602279548	-1.70727234	-2.049498192
2036	8.442964654	12.9961248	-0.403358885	-9.643101543	-1.748094335	-2.065627574
2037	8.440461524	13.06937526	-0.388470273	-9.683923537	-1.788916329	-2.081756956
2038	8.437958394	13.14076526	-0.37358166	-9.724745532	-1.829738324	-2.097886338
2039	8.435455263	13.21029132	-0.358693048	-9.765567526	-1.870560318	-2.11401572
2040	8.432952133	13.27888411	-0.343804435	-9.806389521	-1.911382313	-2.130145101
2041	8.425423867	13.34654276	-0.328915823	-9.847211515	-1.952204307	-2.146274483
2042	8.4178956	13.4123305	-0.31402721	-9.88803351	-1.993026302	-2.162403865
2043	8.410367334	13.47624383	-0.299138598	-9.928855504	-2.033848296	-2.178533247
2044	8.402839067	13.53827922	-0.284249985	-9.969677499	-2.074670291	-2.194662629
2045	8.395310801	13.59843314	-0.269361373	-10.01049949	-2.115492285	-2.210792011
2046	8.382732019	13.65670205	-0.25447276	-10.05132149	-2.15631428	-2.226921393
2047	8.370153237	13.71308238	-0.239584148	-10.09214348	-2.197136274	-2.243050775
2048	8.349950529	13.76662315	-0.224695535	-10.13296548	-2.237958269	-2.259180157
2049	8.337371747	13.81731627	-0.209806923	-10.17378747	-2.278780263	-2.275309539
2050	8.31716904	13.86610643	-0.19491831	-10.21460947	-2.319602258	-2.291438921
